# Novel sulindac derivatives: synthesis, characterisation, evaluation of antioxidant, analgesic, anti-inflammatory, ulcerogenic and COX-2 inhibition activity

**DOI:** 10.1080/14756366.2020.1746783

**Published:** 2020-04-02

**Authors:** Mashooq A. Bhat, Mohamed A. Al-Omar, Nawaf A. Alsaif, Abdulrahman A. Almehizia, Ahmed M. Naglah, Suhail Razak, Azmat Ali Khan, Naeem Mahmood Ashraf

**Affiliations:** a Department of Pharmaceutical Chemistry, College of Pharmacy, King Saud University, Riyadh, Saudi Arabia; b Department of Pharmaceutical Chemistry, Drug Exploration and Development Chair (DEDC), College of Pharmacy, King Saud University, Riyadh, Saudi Arabia; c Peptide Chemistry Department, Chemical Industries Research Division, National Research, Centre, Dokki, Egypt; d Department of Community Health Sciences, College of Applied Medical Sciences, King Saud University, Riyadh, Saudi Arabia; e Department of Biochemistry and Biotechnology, The University of Gujrat, Punjab, Pakistan

**Keywords:** Sulindac hydrazide, hydrazones, antioxidant, analgesic, anti-inflammatory, COX-2 activity

## Abstract

A new series of N′-(substituted phenyl)-2-(1-(4-(methylsulfinyl) benzylidene)−5-fluoro-2-methyl-1*H*-inden-3-yl) acetohydrazide derivatives **(1 – 25)** were prepared in good yields in an efficient manner. All the compounds were fully characterised by the elemental analysis and spectral data. Synthesised compounds were evaluated for antioxidant activity by DPPH method. Compounds **7** (R = 3-methoxyphenyl), **3** (R = 4-dimethylaminophenyl) and **23** (R = 2,4,5-trimethoxy phenyl) substitutions were found to be having highly potent antioxidant activity. Compound **3**, with *para* dimethylaminophenyl substitution was found to be having highest antioxidant activity. It was further evaluated *in vivo* for various analgesic, anti-inflammatory, ulcerogenic and COX-2 inhibitory activity in different animal models. Lead compound **3** was found to be significant anti-inflammatory and analgesic agent. It was also evaluated for ulcerogenic activity and demonstrated significant ulcerogenic reduction activity in ethanol and indomethacin model. The LD_50_ of compound **3** was found to be 131 mg/kg. The animals treated with compound **3** prior to cisplatin treatment resulted in a significant reduction in COX-2 protein expression when compared to cisplatin-treated group. Sulindac derivative with *para* dimethylaminophenyl substitution was found to be the most potent antioxidant, anti-inflammatory and analgesic agent as well as with significant gastric sparing activity as compared to standard drug sulindac. Compound **3** significantly downregulated liver tissue COX‐2 gene expression.

## Introduction

Cyclooxygenase (COX) enzyme catalyses the conversion of arachidonic acid to prostaglandin H_2_ (PGH_2_), which is converted to many prostanoids by specific isomerase enzymes because it is an unstable intermediate. Non-beneficial effects of prostaglandins include pain and fever associated with inflammation; beneficial effects include gastro-intestinal protection and platelet function. The COX-1 and COX-2 are the two isoforms, which are regulated differently. The cyto-protection in the gastrointestinal (GI) tract is provided by COX-1 and COX-2 mediates inflammation^1^.

Non-steroidal anti-inflammatory drugs (NSAIDs) are used to treat pain and inflammation. Side effects include gastrointestinal toxicity such as gastro-duodenal perforations, ulcers and bleeding, ascribed to the inhibition of cyclooxygenase-1 (COX-1). Thus, selective inhibitors of cyclooxygenase-2 (COX-2) were synthesised in an attempt to decrease these side effects^2–6^. Physicians would prescribe gastro-protective agents with a conventional NSAID, prior to the introduction of the COX-2 selective inhibitors. Selectively inhibition of COX-2 enzyme would result in the same anti-inflammatory benefits as that of non-selective NSAIDs provide but with less incidences of gastrointestinal side effects^7^^,^^8^. Some COX-2 inhibitors have also been found to have cardiovascular side effects.

Sulindac is an indene derivative NSAID, known to induce ulceration. New sulindac derivatives are reported as anti-inflammatory^9^, anticancer^10–12^, COX-1 inhibitors^13^ and have shown PPAR γ activity^14^. Syntheses of novel derivatives of NSAIDs have improved their safety profile which resulted in an increased anti-inflammatory activity with reduced ulcerogenicity^15^^,^^16^.

It would be desirable to provide an indene derivative having the anti-inflammatory and analgesic properties of a COX-2 inhibitor NSAID, but which also provides gastric sparing activity. The aim of this study was to prepare novel sulindac acetohydrazide derivatives and evaluate their potential antioxidant, analgesic, anti-inflammatory, ulcerogenic and COX-2 inhibition activity.

## Experimental

### Chemistry

#### Materials and methods

Solvents were procured from Merck. Thin layer chromatography (TLC), was performed on Silica gel 60 F_254_ coated plates (Merck) to check the purity of compounds. For performing FT-IR, Perkin Elmer FT-IR spectrophotometer was used. Melting points were determined by Gallenkamp melting point apparatus. ^1^H and ^13^C NMR were recorded in Bruker NMR 500/700 MHz and 125/176 MHz spectrophotometer. The samples were run in DMSO-*d_6_* with tetra methyl silane (TMS) as an internal standard. Molecular masses of compounds were determined in GC mass spectroscopy. The CHN Elementar (Analysensysteme GmbH, Germany) was used for elemental analysis of the compounds. Cisplatin was purchased from Sigma-Aldrich, USA. Antibodies against COX-2 and β-actin were purchased from Abcam (Cat Log No: ab15191).

#### Synthesis of methyl-5-fluoro-1-{[4-(methane sulfinyl) phenyl] methylidene}-2-methyl-1H-inden-3-yl] acetate

The sulindac ester was prepared according to the reported procedure^17^.

#### 2-[5-Fluoro-1-{[4-(methanesulfinyl)phenyl]methylidene}-2-methyl-1H-inden-3-yl]acetohydrazide

The methyl ester of sulindac (0.01 mol) and hydrazine hydrate (99%) (0.2 mol) were refluxed in methanol (50 ml) for 30 h. The mixture was concentrated, cooled and poured in crushed ice in small portions while stirring, and kept for 3‒4 h at room temperature. The solid separated out was filtered, dried and crystallised from ethanol. The product was carefully checked by thin layer chromatography.

Colour: yellow; Yield: 70%; m.p.: 120‒122 °C; UV λmax (Methanol) = 327 nm; ^1^H NMR (500 MHz, DMSO‒*d_6_*): *δ =*  2.20 (3H, s, CH_3_), 2.82 (3H, s, SOCH_3_), 3.38 (2H, s, CH_2_), 4.28 (2H, bs, NH_2_, D_2_O exchg.), 6.71 (1H, t, *J* = 9.5 Hz, =CH), 7.15–7.80 (7H, m, Ar-H), 9.30 (1H, bs, CONH, D_2_O exchg.); ^13^C NMR (125.76 MHz, DMSO‒*d_6_*): *δ* = 10.88, 31.39, 43.59, 106.74, 106.93, 110.69, 110.87, 123.49, 123.56, 124.39, 129.71, 129.93, 130.40, 133.86, 138.29, 139.07, 140.95, 140.95, 146.69, 147.62, 147.69, 162.63, 163.96, 168.69; MS: *m/z* = 370.44 [M]^+^; Analysis: for C_20_H_19_FN_2_O_2_S, calcd. C 64.85, H 5.17, N 7.56, S 8.66%; found C 64.65, H 5.15, N 7.54 S 8.88%.

#### General procedure for the synthesis of N’-(substituted benzylidene)-2–(1-(4-(methylsulfinyl) benzylidene)-5-fluoro-2-methyl-1H-inden-3-yl) acetohydrazide (1–25)

A solution of sulindac acetohydrazide (1.0 mmol) in ethanol (50 ml) containing appropriate substituted benzaldehydes (1.1 mmol) and a catalytic amount of glacial acetic acid was heated under reflux for 3 h. The reaction mixture was added to the ice cold water in a beaker. The product was precipitated, filtered by vacuum filtration and washed several times with cold water. The solid was recrystallized from ethanol.

#### 2-1-(4-(Methylsulfinyl)benzylidene)-5-fluoro-2-methyl-1H-inden-3-yl)-N′-benzylideneacetohydrazide (1)

Yield: 65%; m. p.: 138‒140 °C; IR (KBr) cm^−1^: 3150 (NH str.), 2980 (CH str.), 1662 (C=O, Str.), 1591 (C=N str.); ^1^H NMR (500 MHz, DMSO‒*d_6_*): *δ* = 2.23 (3H, s, CH_3_), 2.82 (3H, s, S=O CH_3_), 3.59 (1H, s, CH_2_), 4.00 (1H, s, CH_2_), 6.71 (1H, s, =CH), 7.11‒7.78 (12H, m, Ar-H), 8.28 (1H, s, N=CH), 11.50 (1H, s, CONH, D_2_O exchg.); ^13^C NMR (125.76 MHz, DMSO‒*d_6_*): *δ* = 10.9, 29.9, 43.5, 124.3, 127.3, 127.5, 129.0, 129.2, 129.7, 129.9, 130.0, 130.3, 130.4, 134.6, 138.4, 139.0, 140.9, 143.8, 146.6, 171.5; MS: *m/z* = 459.39 [M + 1]^+^; Analysis: for C_27_H_23_FN_2_O_2_S, calcd. C 70.72, H 5.06, N 6.11, S 6.99%; found C 70.99, H 5.05, N 6.14, S 6.97%.

#### N’-(4-Chlorobenzylidene)-2-(1-(4-(methylsulfinyl)benzylidene)-5-fluoro-2-methyl-1H-inden-3-yl)acetohydrazide (2)

Yield: 85%; m. p.: 228‒230 °C; IR (KBr) cm^−1^: 3150 (NH str.), 2955 (CH str.), 1664 (C=O, Str.), 1599 (C=N str.); ^1^H NMR (500 MHz, DMSO‒*d_6_*): *δ* = 2.22 (3H, s, CH_3_), 2.82 (3H, s, S=OCH_3_), 4.00 (2H, s, CH_2_), 6.72 (1H, s, =CH), 7.09‒7.78 (11H, m, Ar-H), 8.00 (1H, s, N=CH), 11.56 (1H, s, CONH, D_2_O exchg.); ^13^C NMR (125.76 MHz, DMSO‒*d_6_*): *δ* = 10.9, 29.9, 43.5, 124.3, 128.9, 129.3, 129.7, 130.4, 134.7, 139.0, 146.7, 171.5; MS: *m/z* = 493.40 [M + 1]^+^; Analysis: for C_27_H_22_ClFN_2_O_2_S, calcd. C 65.78, H 4.50, N 5.68, S 6.50%; found C 65.55, H 4.49, N 6.48, S 6.52%.

#### N′-(4-Dimethylaminobenzylidene)-2-1-(4-(methylsulfinyl)benzylidene)-5-fluoro-2-methyl-1H-inden-3-yl)acetohydrazide (3)

Yield: 50%; m. p.: 158‒160 °C; IR (KBr) cm^−1^: 3165 (NH str.), 3025 (CH str.), 1655 (C=O, Str.), 1599 (C=N str.); ^1^H NMR (500 MHz, DMSO‒*d_6_*): δ = 2.22 (3H, s, CH_3_), 2.82 (3H, s, S=O CH_3_), 2.96 (6H, s, 2 × NCH_3_), 3.54 (1H, s, CH_2_), 3.96 (1H, s, CH_2_), 6.73 (1H, s, =CH), 7.12‒7.78 (11H, m, Ar-H), 7.92 (1H, s, N=CH), 11.19 (1H, s, CONH, D_2_O exchg.); ^13^C NMR (125.76 MHz, DMSO‒*d_6_*): δ = 11.0, 29.9, 43.5, 112.2, 124.3, 128.5, 128.8, 129.6, 129.9, 130.4, 144.7, 146.6, 170.8; MS: *m/z* = 501.77 [M]^+^; Analysis: for C_29_H_28_FN_3_O_2_S, calcd. C 69.44, H 5.63, N 8.38, S 6.39%; found C 69.17, H 5.62, N 8.35, S 6.41%.

#### N′-(3-Hydroxybenzylidene)-2-1-(4-(methylsulfinyl)benzylidene)-5-fluoro-2-methyl-1H-inden-3-yl)acetohydrazide (4)

Yield: 60%; m. p.: 240‒242 °C; IR (KBr) cm^−1^: 3150 (NH str.), 3080 (CH str.), 1664 (C=O, Str.), 1601 (C=N str.); ^1^H NMR (500 MHz, DMSO‒*d_6_*): *δ* = 2.22 (3H, s, CH_3_), 2.81 (3H, s, CH_3_S=O), 3.99 (1H, s, CH_2_), 6.71 (1H, s, =CH), 7.10‒7.78 (11H, m, Ar-H), 7.97 (1H, s, N=CH), 9.65 (1H, s, OH, D_2_O exchg.), 11.44 (1H, s, CONH, D_2_O exchg.); ^13^C NMR (125.76 MHz, DMSO‒*d_6_*): *δ* = 10.9, 29.7, 43.5, 110.7, 113.2, 118.7, 124.3, 129.9, 130.4, 135.8, 140.9, 144.0, 158.1, 171.4; MS: *m/z* = 474.94 [M]^+^; Analysis: for C_27_H_23_FN_2_O_3_S, calcd. C 68.34, H 4.89, N 5.90, S 6.76%; found C 68.07, H 4.90, N 5.88, S 6.78%.

#### N′-(4-Hydroxybenzylidene)-2-(1-(4-(methylsulfinyl)benzylidene)-5-fluoro-2-methyl-1H-inden-3-yl)acetohydrazide (5)

Yield: 50%; m. p.: 210‒212 °C; IR (KBr) cm^−1^: 3165 (NH str.), 3031 (CH str.), 1652 (C=O, Str.), 1597 (C=N str.); ^1^H NMR (500 MHz, DMSO‒*d_6_*): δ = 2.22 (3H, s, CH_3_), 2.83 (3H, s, S=OCH_3_), 3.18 (1H, s, CH_2_), 3.97 (1H, s, CH_2_), 6.72 (1H, s, =CH), 6.82‒7.78 (11H, m, Ar-H), 7.95 (1H, s, N=CH), 9.91 (1H, s, −OH), 11.28 (1H, s, CONH, D_2_O exchg.); ^13^C NMR (125.76 MHz, DMSO‒*d_6_*): δ = 11.0, 29.9, 43.5, 49.0, 106.8, 110.8, 116.1, 124.4, 129.0, 130.4, 144.1, 146.6, 147.4, 147.8, 147.9, 159.6, 159.8, 162.2, 163.6, 165.4, 171.1; MS: *m/z* = 474.75 [M]^+^; Analysis: for C_27_H_23_FN_2_O_3_S, calcd. C 68.34, H 4.89, N 5.90, S 6.76%; found C 68.12, H 4.90, N 5.91, S 6.75%

#### N′-(2-Methoxybenzylidene)-2-(1-(4-(methylsulfinyl) benzylidene)-5-fluoro-2-methyl-1H-inden-3-yl) acetohydrazide (6)

Yield: 55%; m. p.: 173‒176 °C; IR (KBr) cm^−1^: 3150 (NH str.), 3007 (CH str.), 1653 (C=O, Str.), 1564 (C=N str.); ^1^H NMR (500 MHz, DMSO‒*d_6_*): δ = 2.18 (3H, s, CH_3_), 2.80 (3H, s, S=OCH_3_), 3.81 (3H, s, OCH_3_), 3.96 (2H, s, CH_2_), 6.69 (1H, s, =CH), 7.0‒7.77 (11H, m, Ar-H), 8.36 (1H, s, N=CH), 11.45 (1H, s, CONH, D_2_O exchg.); ^13^C NMR (125.76 MHz, DMSO‒*d_6_*): δ = 10.6, 14.7, 31.0, 49.0, 56.2, 112.4, 121.2, 127.0, 133.5, 157.0, 159.2, 171.0; MS: *m/z* = 489.45 [M + 1]^+^; Analysis: for C_28_H_25_FN_2_O_3_S, calcd. C 68.83, H 5.16, N 5.73, S 6.56%; found C 68.56, H 5.15, N 5.75, S 5.13%.

#### N′-(3-Methoxybenzylidene)-2-1-(4-(methylsulfinyl) benzylidene)-5-fluoro-2-methyl-1H-inden-3-yl)acetohydrazide (7)

Yield: 65%; m. p.: 130‒132 °C; IR (KBr) cm^−1^: 3150 (NH str.), 2960 (CH str.), 1664 (C=O, Str.), 1588 (C=N str.); ^1^H NMR (500 MHz, DMSO‒*d_6_*): *δ* = 2.22 (3H, s, CH_3_), 2.82 (3H, s, CH_3_S=O), 3.79 (3H, s, OCH_3_), 4.01 (2H, s, CH_2_), 6.70 (1H, s, =CH), 6.99‒7.78 (11H, m, Ar-H), 8.03 (1H, s, N=CH), 11.52 (1H, s, CONH, D_2_O exchg.); ^13^C NMR (125.76 MHz, DMSO‒*d_6_*): *δ* = 10.9, 11.0, 29.9, 43.5, 55.5, 93.0, 110.8, 116.3, 120.0, 123.5, 124.3, 129.9, 130.4, 133.7, 136.0, 138.4, 138.7, 139.0, 140.9, 143.7, 146.6, 147.1, 160.0, 171.5; MS: *m/z* = 488.85 [M]^+^; Analysis: for C_28_H_25_FN_2_O_3_S, calcd. C 68.83, H 5.16, N 5.73, S 6.56%; found C 69.10, H 5.17, N 5.75, S 6.54%.

#### N′-(2-Nitrobenzylidene)-2-(1-(4-(methylsulfinyl)benzylidene)-5-fluoro-2-methyl-1H-inden-3-yl)acetohydrazide (8)

Yield: 70%; m. p.: 148‒150 °C; IR (KBr) cm^−1^: 3150 (NH str.), 3001 (CH str.), 1665 (C=O, Str.), 1570 (C=N str.); ^1^H NMR (500 MHz, DMSO‒*d_6_*): *δ* = 2.23 (3H, s, CH_3_), 2.82 (3H, s, S=OCH_3_), 3.62 (1H, s, CH_2_), 4.00 (1H, s, CH_2_), 6.71 (1H, s, =CH), 7.06‒8.08 (11H, m, Ar-H), 8.42 (1H, s, N=CH), 11.79 (1H, s, CONH, D_2_O exchg.); ^13^C NMR (125.76 MHz, DMSO‒*d_6_*): *δ* = 10.9, 29.9, 43.5, 124.4, 125.0, 128.7, 129.7, 129.8, 130.4, 130.9, 133.9, 138.5, 139.0, 139.2, 140.8, 142.6, 147.7, 148.4, 171.8; MS: *m/z* = 503.22 [M]^+^; Analysis: for C_27_H_22_FN_3_O_4_S, calcd. C 64.40, H 4.40, N 8.34, S 6.37%; found C 64.60, H 4.41, N 8.36, S 6.39%.

#### N′-(3-Nitrobenzylidene)-2-1-(4-(methylsulfinyl)benzylidene)-5-fluoro-2-methyl-1H-inden-3-yl)acetohydrazide (9)

Yield: 70%; m. p.: 165‒167 °C; IR (KBr) cm^−1^: 3150 (NH str.), 3002 (CH str.), 1654 (C=O, Str.), 1570 (C=N str.); ^1^H NMR (500 MHz, DMSO‒*d_6_*): δ = 2.21 (3H, s, CH_3_), 2.82 (3H, s, S=O CH_3_), 3.63 (1H, s, CH_2_), 4.03 (1H, s, CH_2_), 6.70 (1H, s, =CH), 7.08‒8.24 (11H, m, Ar-H), 8.52 (1H, s, N=CH), 11.73 (1H, s, CONH, D_2_O exchg.); ^13^C NMR (125.76 MHz, DMSO‒*d_6_*): δ = 10.9, 30.0, 43.5, 121.4, 124.3, 124.5, 129.7, 129.8, 130.4, 130.8, 133.4, 136.4, 138.4, 139.0, 140.8, 141.6, 146.6, 148.6, 171.8; MS: *m/z* = 504.41 [M + 1]^+^; Analysis: for C_27_H_23_FN_2_O_2_S, calcd. C 70.72, H 5.06, N 6.11, S 6.99%; found C 71.00, H 5.07, N 6.14, S 6.97%.

#### N′-(4-Nitrobenzylidene)-2-(1-(4-(methylsulfinyl)benzylidene)-5-fluoro-2-methyl-1H-inden-3-yl)acetohydrazide (10)

Yield: 80%; m. p.: 220‒222 °C; IR (KBr) cm^−1^: 3150 (NH str.), 2962 (CH str.), 1666 (C=O, Str.), 1591 (C=N str.); ^1^H NMR (500 MHz, DMSO‒*d_6_*): *δ* = 2.23 (3H, s, CH_3_), 2.82 (3H, s, S=OCH_3_), 3.64 (1H, s, CH_2_), 4.00 (1H, s, CH_2_), 6.71 (1H, s, =CH), 7.08‒7.97 (11H, m, Ar-H), 8.38 (1H, s, N=CH), 11.80 (1H, s, CONH, D_2_O exchg.); ^13^C NMR (125.76 MHz, DMSO‒*d_6_*): *δ* = 10.9, 29.8, 32.0, 43.5, 124.3, 124.4, 128.2, 128.4, 129.8, 130.4, 139.0, 140.8, 141.4, 146.6, 148.1, 166.3, 171.9; MS: *m/z* = 504.28 [M + 1]^+^; Analysis: for C_27_H_22_FN_3_O_4_S, calcd. C 64.40, H 4.40, N 8.34, S 6.37%; found C 64.62, H 4.39, N 8.36, S 6.38%.

#### N′-(2,3-Dihydroxybenzylidene)-2-(1-(4-(methylsulfinyl)benzylidene)-5-fluoro-2-methyl-1H-inden-3-yl)acetohydrazide (11)

Yield: 52%; m. p.: 220‒222 °C; IR (KBr) cm^−1^: 3150 (NH str.), 3060 (CH str.), 1671 (C=O, Str.), 1596 (C=N str.); ^1^H NMR (500 MHz, DMSO‒*d_6_*): δ = 2.24 (3H, s, CH_3_), 2.83 (3H, s, S=OCH_3_), 3.62 (1H, s, CH_2_), 3.97 (1H, s, CH_2_), 6.74 (1H, s, =CH), 6.84‒7.91 (10H, m, Ar-H), 8.42 (1H, s, N=CH), 9.11 (1H, s, OH, D_2_O exchg.), 9.21 (1H, s, OH, D_2_O exchg.), 11.32 (1H, s, CONH, D_2_O exchg.); ^13^C NMR (125.76 MHz, DMSO‒*d_6_*): δ = 10.9, 31.7, 43.5, 110.7, 111.0, 116.9, 117.1, 117.8, 119.1, 119.6, 120.3, 121.2, 124.4, 130.4, 133.1, 133.8, 138.4, 138.9, 139.0, 140.8, 146.0, 146.6, 146.7, 148.3, 165.6, 171.0; MS: *m/z* = 490.70 [M]^+^; Analysis: for C_27_H_23_FN_2_O_4_S, calcd. C 66.11, H 4.73, N 5.71, S 6.54%; found C 66.33, H 5.72, N 5.68, S 6.53%.

#### N′-(2,5-Dihydroxybenzylidene)-2-(1-(4-(methylsulfinyl)benzylidene)-5-fluoro-2-methyl-1H-inden-3-yl)acetohydrazide (12)

Yield: 55%; m. p.: 180‒182 °C; IR (KBr) cm^−1^: 3150 (NH str.), 2915 (CH str.), 1656 (C=O, Str.), 1570 (C=N str.); ^1^H NMR (500 MHz, DMSO‒*d_6_*): δ = 2.22 (3H, s, CH_3_), 2.81 (3H, s, S=OCH_3_), 4.02 (2H, s, CH_2_), 6.72 (1H, s, =CH), 7.18‒7.96 (10H, m, Ar-H), 8.38 (1H, s, N=CH), 8.98 (1H, s, OH, D_2_O exchg.), 10.22 (1H, s, OH, D_2_O exchg.), 11.78 (1H, s, CONH, D_2_O exchg.); ^13^C NMR (125.76 MHz, DMSO‒*d_6_*): δ = 11.0, 29.6, 31.8, 36.2, 106.7, 110.8, 114.0, 117.4, 119.4, 124.4, 129.9, 130.4, 139.0, 140.9, 146.6, 150.3, 162.8, 165.6, 171.0; MS: *m/z* = 490.55 [M]^+^; Analysis: for C_27_H_23_FN_2_O_4_S, calcd. C 66.11, H 4.73, N 5.71, S 6.54%; found C 66.30, H 4.74, N 5.69, S 6.55%.

#### N′-(2,3-Dimethoxybenzylidene)-2-(1-(4-(methylsulfinyl)benzylidene)-5-fluoro-2-methyl-1H-inden-3-yl)acetohydrazide (13)

Yield: 62%; m. p.: 203‒205 °C; IR (KBr) cm^−1^: 3150 (NH str.), 3014 (CH str.), 1654 (C=O, Str.), 1565 (C=N str.); ^1^H NMR (500 MHz, DMSO‒*d_6_*): δ = 2.21 (3H, s, CH_3_), 2.83 (3H, s, S=OCH_3_), 3.84 (1H, s, CH_2_), 3.78 (6H, s, 2 × OCH_3_), 4.00 (1H, s, CH_2_), 6.71 (1H, s, =CH), 7.11‒7.78 (10H, m, Ar-H), 8.35 (1H, s, N=CH), 11.47 (1H, s, CONH, D_2_O exchg.); ^13^C NMR (125.76 MHz, DMSO‒*d_6_*): δ = 10.9, 29.9, 32.0, 43.5, 56.2, 61.6, 106.7, 106.9, 110.7, 114.4, 117.3, 124.3, 130.4, 138.4, 139.0, 139.5, 140.9, 142.5, 146.6, 148.3, 153.1, 162.2, 163.6, 165.7, 171.4; MS: *m/z* = 518.84 [M]^+^; Analysis: for C_29_H_27_FN_2_O_4_S, calcd. C 67.16, H 5.25, N 5.40, S 6.18%; found C 67.3.1, H 5.24, N 5.38, S 6.19%.

#### N′-(2,4-Dimethoxybenzylidene)-2-(1-(4-(methylsulfinyl)benzylidene)-5-fluoro-2-methyl-1H-inden-3-yl)acetohydrazide (14)

Yield: 60%; m. p.: 185‒187 °C; IR (KBr) cm^−1^: 3150 (NH str.), 3011 (CH str.), 1654 (C=O, Str.), 1600 (C=N str.); ^1^H NMR (500 MHz, DMSO‒*d_6_*): δ = 2.21 (3H, s, CH_3_), 2.84 (3H, s, S=OCH_3_), 3.83 (6H, s, 2 × CH_3_), 3.84 (2H, s, CH_2_), 6.63 (1H, s, =CH), 7.13‒7.74 (10H, m, Ar-H), 8.32 (1H, s, N=CH), 11.32 (1H, s, CONH, D_2_O exchg.); ^13^C NMR (125.76 MHz, DMSO‒*d_6_*): δ = 10.9, 29.9, 32.0, 43.5, 55.8, 56.1, 98.5, 106.7, 110.7, 115.3, 123.4, 124.3, 127.0, 129.5, 130.3, 133.4, 138.2, 139.0, 140.8, 142.6, 147.5, 159.4, 162.2, 163.6, 165.4, 171.1; MS: *m/z* = 518.91 [M]^+^; Analysis: for C_29_H_27_FN_2_O_4_S, calcd. C 67.16, H 5.25, N 5.40, S 6.18%; found C 67.33, H 5.26, N 5.42, S 6.17%.

#### N′-(3,4-Dimethoxybenzylidene)-2-(1-(4-(methylsulfinyl)benzylidene)-5-fluoro-2-methyl-1H-inden-3-yl)acetohydrazide (15)

Yield: 65%; m. p.: 230‒232 °C; IR (KBr) cm^−1^: 3150 (NH str.), 3002 (CH str.), 1656 (C=O, Str.), 1571 (C=N str.); ^1^H NMR (500 MHz, DMSO‒*d_6_*): δ = 2.25 (3H, s, CH_3_), 2.83 (3H, s, S=OCH_3_), 3.58 (1H, s, CH_2_), 3.81 (6H, s, 2 × OCH_3_), 4.00 (1H, s, CH_2_), 6.72 (1H, s, =CH), 7.01‒7.79 (10H, m, Ar-H), 7.80 (1H, s, N=CH), 11.38 (1H, s, CONH, D_2_O exchg.); ^13^C NMR (125.76 MHz, DMSO‒*d_6_*): δ = 10.9, 30.0, 32.0, 43.5, 55.8, 56.0, 108.7, 111.9, 121.8, 122.2, 124.4, 127.3, 129.8, 130.4, 138.3, 139.4, 138.3, 139.0, 140.8, 144.0, 146.6, 149.4, 150.9, 171.2; MS: *m/z* = 518.58 [M]^+^; Analysis: for C_29_H_27_FN_2_O_4_S, calcd. C 67.16, H 5.25, N 5.40, S 6.18%; found C 67.32, H 5.26, N 5.38, S 6.17%.

#### N′-(2-Hydroxy-3-methoxymethoxybenzylidene)-2-(1-(4-(methylsulfinyl)benzylidene)-5-fluoro-2-methyl-1H-inden-3-yl)acetohydrazide (16)

Yield: 55%; m. p.: 210‒212 °C; IR (KBr) cm^−1^: 3180 (NH str.), 3051 (CH str.), 1693 (C=O, Str.), 1599 (C=N str.); ^1^H NMR (500 MHz, DMSO‒*d_6_*): δ = 2.24 (3H, s, CH_3_), 2.83 (3H, s, S=OCH_3_), 3.61 (1H, s, CH_2_), 3.80 (3H, s, OCH_3_), 4.00 (1H, s, CH_2_), 6.82 (1H, s, =CH), 7.01‒7.79 (10 H, m, Ar-H), 8.48 (1H, s, N=CH), 10.78 (1H, s, OH, D_2_O exchg.), 11.85 (1H, s, CONH, D_2_O exchg.); ^13^C NMR (125.76 MHz, DMSO‒*d_6_*): δ = 10.9, 29.9, 31.7, 43.5, 56.2, 106.7, 113.2, 114.1, 118.0, 119.3, 119.4, 119.6, 121.3, 130.4, 138.3, 138.8, 138.9, 139.0, 140.8, 141.0, 147.4, 162.2, 162.3, 163.7, 165.6, 171.2; MS: *m/z* = 504.34 [M]^+^; Analysis: for C_28_H_25_FN_2_O_4_S, calcd. C 66.65, H 4.99, N 5.55, S 6.35%; found C 66.85, H 4.50, N 5.53, S 6.34%.

#### N′-(3-Hydroxy-4-methoxybenzylidene)-2-(1-(4-(methylsulfinyl)benzylidene)-5-fluoro-2-methyl-1H-inden-3-yl)acetohydrazide (17)

Yield: 58%; m. p.: 208‒210 °C; IR (KBr) cm^−1^: 3150 (NH str.), 3023 (CH str.), 1657 (C=O, Str.), 1569 (C=N str.); ^1^H NMR (500 MHz, DMSO‒*d_6_*): δ = 2.22 (3H, s, CH_3_), 2.83 (3H, s, S=OCH_3_), 3.56 (1H, s, CH_2_), 3.82 (3H, s, OCH_3_), 3.99 (1H, s, CH_2_), 6.72 (1H, s, =CH), 6.98‒7.80 (10H, m, Ar-H), 7.92 (1H, s, N=CH), 9.24 (1H, s, OH, D_2_O exchg.), 11.32 (1H, s, CONH, D_2_O exchg.); ^13^C NMR (125.76 MHz, DMSO‒*d_6_*): δ = 10.9, 29.7, 32.0, 43.5, 56.0, 106.7, 112.6, 120.3, 120.7, 127.4, 130.4, 138.4, 138.6, 139.0, 140.8, 144.1, 147.2, 150.2, 162.2, 163.6, 165.5, 171.1; MS: *m/z* = 504.94 [M]^+^; Analysis: for C_28_H_25_FN_2_O_4_S, calcd. C 66.65, H 4.99, N 5.55, S 6.35%; found C 66.45, H 4.98, N 5.56, S 6.36%.

#### N′-(3-Ethoxy-4-hydroxybenzylidene)-2-(1-(4-(methylsulfinyl) benzylidene)-5-fluoro-2-methyl-1H-inden-3-yl)acetohydrazide (18)

Yield: 60%; m. p.: 193‒195 °C; IR (KBr) cm^−1^: 3255 (NH str.), 2940 (CH str.), 1661 (C=O, Str.), 1592 (C=N str.); ^1^H NMR (500 MHz, DMSO‒*d_6_*): δ = 1.37 (3H, s, CH_3_), 2.21 (3H, s, CH_3_), 2.83 (3H, s, S=OCH_3_), 3.43 (2H, q, OCH_2_), 4.04 (2H, s, CH_2_), 6.83 (1H, s, =CH), 7.17‒7.81 (10H, m, Ar-H), 8.16 (1H, s, N=CH), 9.49 (1H, s, OH, D_2_O exchg.), 11.31 (1H, s, CONH, D_2_O exchg.); ^13^C NMR (125.76 MHz, DMSO‒*d_6_*): δ = 11.0, 15.2, 30.1, 43.6, 54.3, 110.9, 116.0, 121.9, 123.6, 124.4, 126.0, 129.8, 130.0, 133.8, 138.3, 138.7, 139.1, 140.9, 144.4, 146.5, 147.6, 149.4, 162.3, 163.7, 165.6, 171.2; MS: *m/z* = 520.49 [M + 2]^+^; Analysis: for C_29_H_27_FN_2_O_4_S, C 67.16, H 5.25, N 5.40, S 6.18%; found C 67.35, H 5.24, N 5.42, S 6.19%.

#### N′-(3-Methoxy-4-ethoxybenzylidene)-2-(1-(4-(methylsulfinyl)benzylidene)-5-fluoro-2-methyl-1H-inden-3-yl)acetohydrazide (19)

Yield: 65%; m. p.: 158‒160 °C; IR (KBr) cm^−1^: 3227 (NH str.), 3100 (CH str.), 1658 (C=O, Str.), 1603 (C=N str.); ^1^H NMR (500 MHz, DMSO‒*d_6_*): δ = 1.32 (3H, s, CH_3_), 2.22 (3H, s, CH_3_), 2.80 (3H, s, S=OCH_3_), 3.76 (3H, s, OCH_3_), 3.98 (2H, q, OCH_2_), 4.04 (1H, s, CH_2_), 6.71 (1H, s, =CH), 6.96‒7.95 (10H, m, Ar-H), 8.17 (1H, s, N=CH), 11.36 (1H, s, CONH, D_2_O exchg.); ^13^C NMR (125.76 MHz, DMSO‒*d_6_*): δ = 10.9, 11.0, 15.1, 30.0, 43.5, 55.7, 64.1, 106.8, 108.7, 110.7, 120.6, 121.7, 124.4, 127.1, 129.9, 130.4, 133.7, 138.3, 138.7, 139.0, 140.9, 144.0, 146.6, 147.4, 147.8, 149.5, 150.3, 162.2, 163.6, 165.6, 171.2; MS: *m/z* = 535.19 [M + 2]^+^; Analysis: for C_30_H_29_FN_2_O_4_S, calcd. C 67.65, H 5.49, N 5.26, S 6.02%; found C 67.42, H 5.50, N 5.24, S 6.01%.

#### N′-(3,5-Dimethoxy-4-hydroxybenzylidene)-2-(1-(4-(methylsulfinyl)benzylidene)-5-fluoro-2-methyl-1H-inden-3-yl)acetohydrazide (20)

Yield: 65%; m. p.: 218‒220 °C; IR (KBr) cm^−1^: 3150 (NH str.), 3010 (CH str.), 1654 (C=O, Str.), 1577 (C=N str.); ^1^H NMR (500 MHz, DMSO‒*d_6_*): δ = 2.23 (3H, s, CH_3_), 2.82 (3H, s, S=OCH_3_), 3.84 (6H, s, 2 × OCH_3_), 3.98 (2H, s, CH_2_), 6.74 (1H, s, =CH), 7.17‒7.80 (9H, m, Ar-H), 8.33 (1H, s, N=CH), 11.31 (1H, s, CONH, D_2_O exchg.); ^13^C NMR (125.76 MHz, DMSO‒*d_6_*): δ = 10.9, 30.1, 43.5, 56.2, 56.9, 98.2, 106.8, 107.9, 108.3, 110.7, 113.6, 124.3, 130.4, 138.3, 139.0, 140.8, 142.7, 143.6, 146.6, 152.2, 153.6, 165.3, 171.1; MS: *m/z* = 534.59 [M]^+^; Analysis: for C_29_H_27_FN_2_O_5_S, calcd. C 65.15, H 5.09, N 5.24, S 6.00%; found C 65.30, H 5.10, N 5.22, S 6.01%.

#### N′-(2,3,4-Trihydroxybenzylidene)-2-(1-(4-(methylsulfinyl)benzylidene)-5-fluoro-2-methyl-1H-inden-3-yl)acetohydrazide (21)

Yield: 50%; m. p.: 195‒197 °C; IR (KBr) cm^−1^: 3180 (NH str.), 3051 (CH str.), 1668 (C=O, Str.), 1598 (C=N str.); ^1^H NMR (500 MHz, DMSO‒*d_6_*): *δ* = 2.24 (3H, s, CH_3_), 2.83 (3H, s, S=OCH_3_), 3.59 (1H, s, CH_2_), 6.38 (1H, s, =CH), 6.71‒7.81 (10 H, m, Ar-H), 8.30 (1H, s, N=CH), 9.24 (1H, s, OH, D_2_O exchg.), 11.29 (1H, s, OH, D_2_O exchg.), 11.73 (1H, s, CONH, D_2_O exchg.); ^13^C NMR (125.76 MHz, DMSO‒*d_6_*): *δ* = 10.9, 29.7, 31.7, 43.5, 108.0, 111.1, 121.5, 124.4, 130.4, 133.1, 138.8, 139.0, 140.8, 146.7, 147.8, 149.2, 149.5, 162.3, 163.7, 165.2, 170.5; MS: *m/z* = 504.94 [M-1]^+^; Analysis: for C_27_H_23_FN_2_O_5_S, calcd. C 64.02, H 4.58, N 5.53, S 6.33%; found C 64.19, H 4.57, N 5.55, S6.31%.

#### N′-(2,3,4-Timethoxybenzylidene)-2-(1-(4-(methylsulfinyl)benzylidene)-5-fluoro-2-methyl-1H-inden-3-yl)acetohydrazide (22)

Yield: 70%; m. p.: 185‒187 °C; IR (KBr) cm^−1^: 3150 (NH str.), 2987 (CH str.), 1651 (C=O, Str.), 1589 (C=N str.); ^1^H NMR (500 MHz, DMSO‒*d_6_*): δ = 2.21 (3H, s, CH_3_), 2.82 (3H, s, S=OCH_3_), 3.82 (9H, s, 3 × OCH_3_), 3.97 (2H, s, CH_2_), 6.73 (1H, s, =CH), 7.18‒7.79 (9H, m, Ar-H), 8.24 (1H, s, N=CH), 11.35 (1H, s, CONH, D_2_O exchg.);^13^C NMR (125.76 MHz, DMSO‒*d_6_*): δ = 11.0, 30.0, 32.1, 43.6, 56.5, 61.0, 62.3, 106.9, 109.2, 110.9, 121.0, 124.5, 130.0, 130.5, 138.4, 139.1, 139.8, 141.0, 142.1, 142.6, 146.7, 153.0, 155.5, 162.3, 163.7, 165.6, 171.3; MS: *m/z* = 548.03 [M]^+^; Analysis: for C_30_H_29_FN_2_O_5_S, calcd. C 65.68, H 5.33, N 5.11, S 5.84%; found C 65.45, H 5.34, N 5.13, S 5.85%.

#### N′-(2,4,5-Trimethoxybenzylidene)-2-1-(4-(methylsulfinyl)benzylidene)-5-fluoro-2-methyl-1H-inden-3-yl)acetohydrazide (23)

Yield: 60%; m. p.: 233‒235 °C; IR (KBr) cm^−1^: 3150 (NH str.), 2914 (CH str.), 1658 (C=O, Str.), 1598 (C=N str.); ^1^H NMR (500 MHz, DMSO‒*d_6_*): δ = 2.16 (3H, s, CH_3_), 2.82 (3H, s, S=OCH_3_), 3.63 (9H, s, 3 × OCH_3_), 3.70 (2H, s, CH_2_), 6.73 (1H, s, =CH), 7.01‒7.79 (9H, m, Ar-H), 8.80 (1H, s, N=CH), 11.11 (1H, s, CONH, D_2_O exchg.); ^13^C NMR (125.76 MHz, DMSO‒*d_6_*): δ = 10.70, 31.0, 43.5, 52.3, 106.4, 110.9, 124.4, 130.4, 138.8, 140.6, 147.2, 160.3, 162.3, 162.6, 163.6, 171.0; MS: *m/z* = 544.67 [M-4]^+^; Analysis: for C_30_H_29_FN_2_O_5_S, calcd. C 65.68, H 5.33, N 5.11, S 5.84%; found C 65.45, H 5.32, N 5.09, S 5.85%.

#### N′-(2,4,6-Timethoxybenzylidene)-2-(1-(4-(methylsulfinyl)benzylidene)-5-fluoro-2-methyl-1H-inden-3-yl)acetohydrazide (24)

Yield: 68%; m. p.: 193‒195 °C; IR (KBr) cm^−1^: 3150 (NH str.), 3001 (CH str.), 1652 (C=O, Str.), 1584 (C=N str.); ^1^H NMR (500 MHz, DMSO‒*d_6_*): δ = 2.19 (3H, s, CH_3_), 2.79 (3H, s, S=OCH_3_), 3.78 (9H, s, 3 × OCH_3_), 3.85 (2H, s, CH_2_), 6.68 (1H, s, =CH), 7.18‒7.72 (9H, m, Ar-H), 8.22 (1H, s, N=CH), 11.11 (1H, s, CONH, D_2_O exchg.); ^13^C NMR (125.76 MHz, DMSO‒*d_6_*): δ = 10.86, 29.38, 32.0, 43.5, 55.8, 56.3, 91.5, 104.1, 106.8, 110.8, 123.5, 124.4, 129.6, 130.4, 138.6, 139.1, 141.0, 142.9, 146.5, 147.9, 160.3, 162.3, 162.7, 163.7, 171.1; MS: *m/z* = 547.97 [M-1]^+^; Analysis: for C_30_H_29_FN_2_O_5_S, calcd. C 65.68, H 5.33, N 5.11, S 5.84%; found C 65.88, H 5.34, N 5.13, S 5.85%.

#### N′-(3,4,5-Trimethoxybenzylidene)-2-(1-(4-(methylsulfinyl)benzylidene)-5-fluoro-2-methyl-1H-inden-3-yl)acetohydrazide (25)

Yield: 62%; m. p.: 165‒167 °C; IR (KBr) cm^−1^: 3150 (NH str.), 2917 (CH str.), 1652 (C=O, Str.), 1598 (C=N str.); ^1^H NMR (500 MHz, DMSO‒*d_6_*): δ = 2.23 (3H, s, CH_3_), 2.82 (3H, s, S=OCH_3_), 3.81 (9H, s, 3 × OCH_3_), 4.01 (1H, s, CH_2_), 6.72 (1H, s, =CH), 7.05‒7.79 (9H, m, Ar-H), 7.98 (1H, s, N=CH), 11.50 (1H, s, CONH, D_2_O exchg.); ^13^C NMR (125.76 MHz, DMSO‒*d_6_*): δ = 11.2, 30.2, 43.6, 56.4, 60.7, 104.6, 107.0, 110.9, 123.6, 124.5, 130.0, 130.5, 133.8, 138.8, 139.1, 139.5, 140.9, 143.9, 146.7, 147.3, 147.7, 153.7, 162.4, 163.7, 171.6; MS: *m/z* = 547.64 [M-1]^+^; Analysis: for C_30_H_29_FN_2_O_5_S, calcd. C 65.68, H 5.33, N 5.11, S 5.84%; found C 65.43, H 5.32, N 5.13, S 5.82%.

#### DPPH radical scavenging assay

The antioxidant activity was measured based on the scavenging activity of the stable DPPH free radical. The antioxidant activity was determined by following the method^18^. The compounds in concentration of (100 µg/mL) were added to 3 ml of 0.004% DPPH solution. Methanol was replaced in the control sample. Absorbance was determined at 520 nm after 30 min. Butylated hydroxytoluene (BHT) was used a reference drug. The percent inhibition was calculated by the following equation: *A*_0_ − *A*_t_/*A*_o_ × 100, where *A*_t_ = absorbance of compound, *A*_0_ = absorbance of control.

#### Pharmacological activities

Thirty-five Adult wistar male rats weighing 240–260 g, 12–14 weeks’ old and mice were obtained from animal house of Department of the Community Health Sciences, College of Applied Medical Sciences, King Saud University, Riyadh, KSA. Animals were given favourable conditions (temperature 25 °C, 12/12 h light and dark cycle, and humidity 60 ± 10% and pathogen-free environment). The rats were fed a dietary formulation of protein (18.1%), fat (7.1%), carbohydrate (59.3%) and fibre (15.5%) with food and water being provided *ad libitum*. The study protocol was approved by Ethical Committee of College of Applied Medical Sciences, King Saud University, Saudi Arabia. (Ethics Number: CAMS 22 – 39/40).

#### Anti-pyretic study

Hyperthermia was induced in mice by (*s.c.*) injection of (20 ml/kg) of a 20% aqueous suspension of brewer’s yeast in the back below of nape of the neck^19^. The animals were fasted for the duration of 24 h. Water was made available. Control temperature was taken 24 h after the yeast injection to determine the pyretic response to yeast. Temperature taken 1 h prior to drug administration in the fevered animals, served as a pre drug control. Drugs were given 24 h after the yeast injection and temperature were recorded at 60, 90 and 120 min. after the administration.

#### Analgesic study by tail flick method

Acute nociception was assessed using a tail flick apparatus (Tail Flick model DS 20 Sorrel Apelex, France) following the method^20^. Briefly, each animal was placed in a restrainer, 2 min. before treatment, and baseline reaction time was measured by focussing an intensity controlled beam of light on the distal one-third portion of the animals’ tail. The suspension was orally administered immediately after this step and 25 min. later, the post drug reaction time was measured. A 10-s cut-off time was used in order to prevent tissue damage.

#### Analgesic activity by hot plate method

The hot plate method used as described by Turner^21^. The animals were dropped gently on a hot plate maintained at 55 ± 5.5 °C. The reaction time was taken as the interval extending from the instant the animal reached the hot plate until the moment the animal licked its forefeet or jumped off. The reaction time was measured 10 min before the oral administration of the drug and +60 + 90 + 120 min after treatments.

#### Analgesic activity by writhing test

Writhing was induced in mice by intraperitoneal administration of 0.1 ml of 1% acetic acid. The number of writhing movements was counted for 20 min. The writhing test was performed after the administration of the vehicle or drug.

#### Carrageenan-induced paw edoema in rats

Pedal inflammation in albino rats of either sex was produced according to the reported method^22^. An injection wad made of 0.05 ml of 1% carrageenan sodium salt (BDH) into right hind foot of each rat under the plantar aponeurosis. The test group of rats was treated orally with drugs 1 h before carrageenan injection. At the same time, control group was given 5 ml/kg of normal saline and the reference group was given 100 mg/kg of an aqueous solution of sulindac. The measurements of foot volume were done by the displacement technique using a plethysmometer (Apelex, France) immediately after and +2 and +3 h after the injection of carrageenan. The inhibitory activity was calculated to the following formula 100 (1 − a − x/b − y); where “b” is the mean paw volume of control rats after carrageenan injection and “y” before the injection; whereas” x” is the mean paw volume of treated rats before injection and “a” is the mean paw volume after carrageenan injection.

#### Ulcer study of drugs using 80% ethanol

The ethanol induced ulcer model was used to study gastro-protective activity of compound **3**. The rats were grouped into five groups (*n* = 6). Groups I and II received saline solution and served as negative-control and ulcer-control, respectively. Group III received compound **3** (150 mg/kg) orally and served as the experimental drug group. Animals in groups IV received the Sulindac (100 mg/kg body weight). After 1 h, all of the groups, except Group I, received (20 ml/kg) of 80%. The animals were sacrificed 1 h later under anaesthesia and their stomachs were quickly removed for further studies^23^.

#### Gastric lesions induced by indomethacin

Suspension of Indomethacin in 1.0% carboxymethylcellulose (CMC) in water (6 mg/mL) at a dose of (30 mg/kg) body weight was administered orally. Control rats were treated with vehicle. Compound **3** was given half an hour prior to Indomethacin administration at a dose of 150 mg/kg^24^.

#### Determination of malondialdehyde (MDA)

The MDA was measured according to the method by Utley and others^25^. The tissue was removed and each tissue was homogenised in 0.15 M KCl to give a 10% W/v homogenate. Aliquots of homogenate (1 ml) were incubated at 37 °C for 3 h in a metabolic shaker. Then 1 ml of 10% aqueos trichloroacetic acid was added and mixed. This was then centrifuged at 4000 rpm for 10 min. Total of 1 ml of the supernatant was removed and mixed with 1 ml of 0.67% thiobarbituric acid in water and placed in a boiling water bath for 10 min. The mixture was cooled and diluted with 1 ml of distilled water. The absorbance of the solution was then read at 535 nm. The content of MDA (nmol/g) was then calculated, by the reference to a standard curve of MDA solution.

#### Estimation of non-protein sulfhydryls (NP-SH)

Hepatic non-protein sulfhydryls (NP-SH) was measured according to the reported method by Sedlak and Lindsay^26^. The tissue was homogenised in ice cold 0.02 mmol/L ethylenediaminetetraacetic acid (EDTA). The Aliquotes of 5 ml of the homogenates were mixed in 15 ml test tube with 4 ml of distilled water and 1 ml of 50% trichloacetic acid (TCA). The tube was shaken intermittently for 10 min. and centrifuged 3000 rpm for 10 min. Total of 2 ml supernatant was mixed with 4 ml of 0.4 mmol/L tris buffer (pH 8.9). Total of 0.1 ml of 5,5-dithiobis (2-nitrobenzoic acid) (BTNB) was added and the sample was shaken. The absorbance was measured within 5 min of addition of DTNB at 412 nm against reagent blank.

#### Determination of LD_50_

The LD_50_ (lethal dose 50%) was calculated for compound **3** by Karber method^27^. For determination of LD_50_, an observation was made for 24 h and symptoms of toxicity and rate of mortality were noted. Expired animals were counted at the end of the study period for the calculation of LD_50_. LD_50_ = LD_100_ − ∑ × (a × b)/n, where n is the total number of animals in a group, a is the difference between two successive doses of administered extract/substance, b is the average number of dead animals in two successive doses, and LD_100_ is the lethal dose causing 100% death of all test animals.

#### COX-2 mRNA expression in cisplatin-induced hepatotoxicity in rats

The animals were divided into five groups and each group with seven rats.

- Group 1: Control (Normal) group, received a single dose (i.p) of isotonic saline on the second day of experiment.
- Group 2: DMSO group, received a single dose (i.p) of 2% DMSO on the second day of experiment.
- Group 3: Cisplatin group, received a single dose of cisplatin (12 mg/kg, i.p) on the second day of experiment.
- Group 4: Cisplatin – compound **3** group, received compound **3** (20 mg/kg i.p) for 7 days and a single dose of Cisplatin (12 mg/kg) on the second day of experiment, 1 h after the dose of compound **3**.
- Group 5: Cisplatin – compound **3** group, received compound **3** (40 mg/kg i.p) for 7 days and a single dose of Cisplatin (12 mg/kg) on the second day of experiment, 1 h after the dose of compound 8.

The doses of compound **3** and cisplatin were selected after performing the pilot experiment.

#### Sample collection and preparation

On the last day of experiment, all animals were terminated and anaesthesia was made by injecting ketamine/xylazine mixture (75/2.5 mg/kg, respectively) via the intraperitoneal route. Anaesthetised rats were secured in a supine position and organ samples were taken from the liver. The liver tissues were quickly harvested. The tissues were treated with liquid nitrogen and were used for RNA extraction and immunoblotting.

#### Extraction of protein and Western blot analysis

The previously developed procedures with slight modifications were used to perform SDS-PAGE and western blot investigations. The protein concentration was estimated by Bradford assay. For western blotting 8–12% polyacrylamide gels were used to resolve 40 μM of protein, transferred on to a nitrocellulose membrane, probed with appropriate monoclonal primary antibodies, and detected by super signal west Pico, Dura or Femto Chemiluminescence Reagent (Thermo Scientific, USA). Quantification of protein bands was done through measuring band density using Image J software. The densities of the bands (normalised to actin) relative to that of the untreated control (designated as 1.00) were presented as mean ± SEM of three separate experiments.

#### Gene expression analysis

Total RNA from frozen liver was extracted by using kit, according to the manufacturer’s instructions (Promega, CatLog No: Z3101). The cDNA synthesis was performed using the Applied Biosystems™ High-Capacity cDNA Reverse Transcription Kit. The reaction mixture was prepared containing 10 µL FastStart Universal SYBR Green Master (Roche, Germany), 6 µM reverse primers, and 10 µg cDNA, with RNAase free water added to a total volume of 20 µL. The amplification and real-time analysis were done for 40 cycles with following factors; 95 °C (10 min.) in order to activate of FastStart Taq DNA polymerase; 60 °C (1 min.) for amplification and real-time analysis. The gene expression levels were determined using 2-ΔΔCT. Primer sequences used are shown below:

#### Primers

Candidate gene primer

- COX-2 F: 5′-CACTCATGAGCAGTCCCCTC-3′
- R: 5′-ACCCTGGTCGGTTTGATGTT-3′

#### Molecular docking of compounds against COX-2 protein

Three-dimensional structure of the Cox-2 gene was developed using homology modelling. Modeller 9.17 was employed to predict the structure using templates (5F1A, 5IKQ, 5F19) downloaded from PDB. All of the models showed more than 90% identity with our protein. The predicted structure was further refined by energy minimisation. Finally, the structure was validated using the Ramachandran plot. Furthermore, three-dimensional structures of all synthetic compounds and Sulindac were constructed using Chem 3 D Pro 12.0 version. The protein-ligand docking analysis was performed using online PatchDock server. Three-dimensional structures of protein and ligands were used as input. PatchDock server rated the all possible docking confirmations using minimum ACE (Atomic contact energies). Finally, the docking confirmations were visualised using Pymol and LigPlus.

## Results and discussion

### Chemistry

**Scheme 1.**
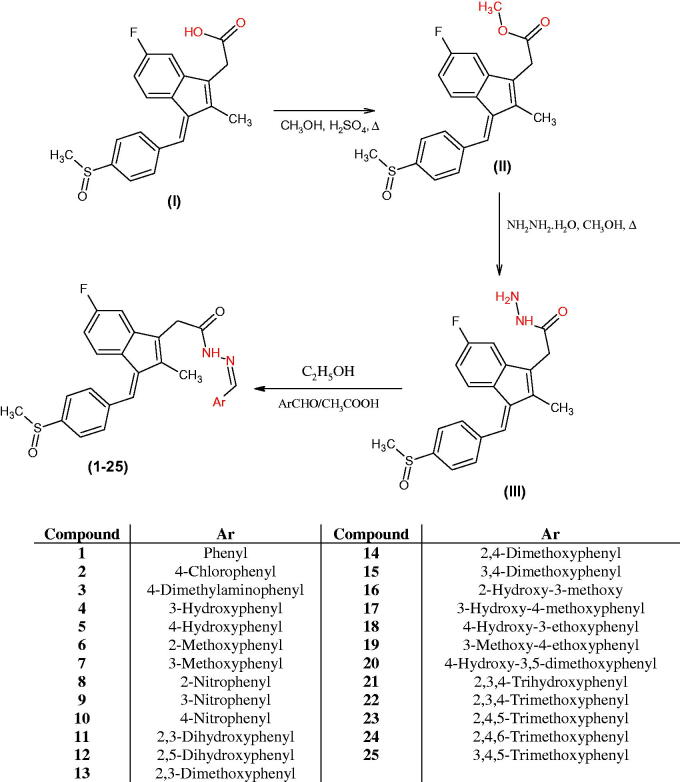
Synthetic route of compounds **(1‒25)**.

Scheme 1 illustrates the synthesis of the acetohydrazide derivatives **(1 – 25)**. The compound acetohydrazide **(III)** was synthesised by refluxing methyl ester of sulindac and hydrazine hydrate (99%) in the presence of absolute ethanol. Sulindac methyl ester **(II)** was synthesised from sulindac by refluxing in methanol with concentrated sulphuric acid according to the reported procedure.

The acetohydrazide **(III)** was used as a starting material for the synthesis of various substituted sulindac hydrazone derivatives (**1 – 25**). The acetohydrazide **(III)** was reacted substituted benzaldehydes in ethanol and glacial acetic acid as a catalyst. The acetohydrazide was characterised by the appearance of singlet peak for the –NH_2_ protons at δ 3.38 ppm and broad singlet for the CONH proton at δ 9.30 ppm. The disappearance of NH_2_ protons at δ 3.38 ppm confirmed the structures of sulindac hydrazones. Carbon-13 NMR confirmed all the carbon atoms of the synthesised compounds (**1‒25**). Mass spectroscopy confirmed the molecular weights of compounds. All the compounds were characterised by their molecular ion peaks. The three methyl protons of the indene moiety appeared in the range of δ 2.18‒2.23 ppm. The three protons of –SOCH_3_ appear at δ 2.81‒2.83 ppm. The aromatic protons appeared in the range of δ 6.71‒8.24 ppm. Protons of the N=CH appeared as a singlet in the range of δ 7.92‒8.52 ppm and the proton of CONH appeared as broad singlet at δ 9.30‒11.80 ppm.

### Antioxidant activity

**Figure 1.**
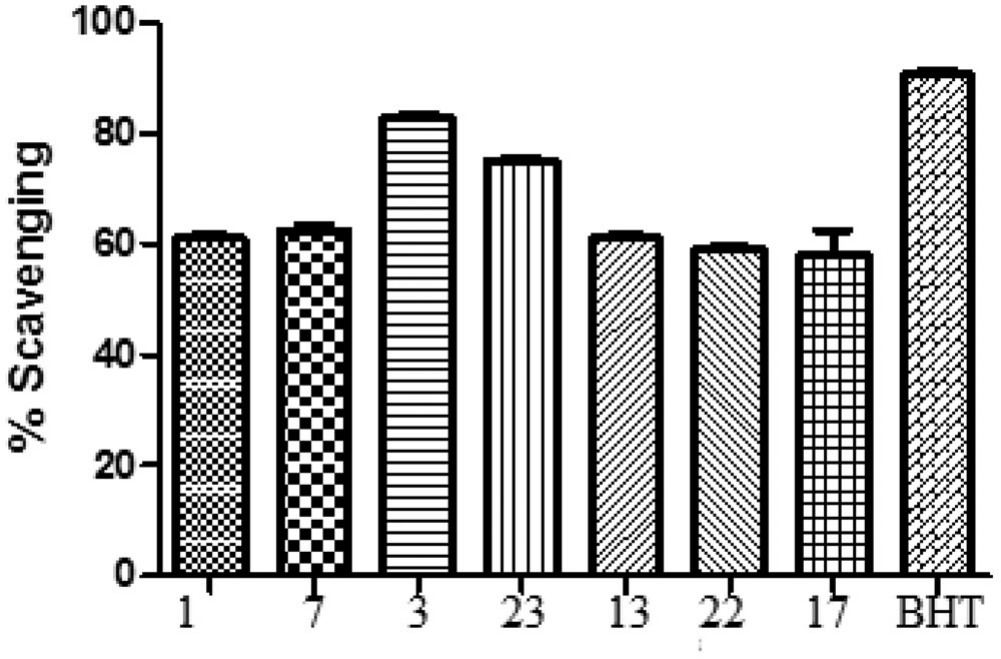
Comparison of DPPH scavenging activity of compounds **(1–25)** and BHT. All values are means of three replicates ± SD.

The synthesised compounds **(1‒25)** were evaluated for their antioxidant activity by DPPH method (Figure 1). All the compounds exhibit antioxidant activity from (18.53 ± 4.86) to (85.10 ± 6.80) as compared to standard drug BHT (90.6 ± 3.83) (Table 1).

**Table 1.** Antioxidant activity of compounds **(1–25)** by DPPH method.

Compound	Scavenging effect % (mean ± SD)^a^
**1**	67.40 ± 9.25
**2**	39.86 ± 10.80
**3**	**85.10 ± 6.80**
**4**	42.20 ± 13.20
**5**	45.06 ± 22.57
**6**	39.73 ± 15.65
**7**	71.03 ± 5.31
**8**	18.53 ± 4.86
**9**	49.36 ± 5.57
**10**	19.26 ± 5.32
**11**	33.10 ± 8.41
**12**	38.10 ± 10.92
**13**	62.93 ± 6.72
**14**	23.07 ± 6.95
**15**	40.60 ± 14.40
**16**	27.66 ± 2.12
**17**	51.40 ± 16.66
**18**	35.50 ± 9.05
**19**	37.90 ± 10.35
**20**	29.66 ± 16.93
**21**	30.06 ± 3.30
**22**	63.56 ± 0.47
**23**	78.97 ± 9.36
**24**	30.80 ± 2.50
**25**	42.33 ± 18.38
BHT	90.6 ± 3.83

Compound number 3 having highest Scavenging effect represented in bold.

^a^All values are means of three replicates ± SD.

### Structure–activity relationship (SAR)

Compound **3** (R = *para* dimethylaminophenyl) was found to be highly potent antioxidant (85.10 ± 6.80). Compounds **1** (R = phenyl), **7** (R = 3-methoxyphenyl), **23** (R = 2,4,6-trimethoxyphenyl), **13** (R = 2,3,dimethoxyphenyl), **22** (R = 2,3,4-trimethoxyphenyl) and **17** (R = 3-hydroxy, 4-methoxy) were found to be significantly active as antioxidants as compared to BHT. Compounds containing hydroxyl phenyl substitutions were found to be week antioxidants. Compounds **8** (R = 2-nitrophenyl) and **10** (R = 4-nitrophenyl) were found to be least active as antioxidants. Electron donating groups like methoxy, were found to have positive effect while as electron withdrawing groups like nitro, was found to have negative effect on antioxidant activity. Compound **3** having *para* dimethylaminophenyl group was found to be the most potent anti-oxidant compound.

### Analgesic activity

#### Tail flick method

Tail flick method was used for testing the analgesic activity of compound **3**. After 30 min, the % inhibition of test compound **3** was 3.33% as compared to reference drug sulindac with 13.79%. The testing compound **3** expressed significant activity of 46.66% inhibition compared to reference drug sulindac with 82.75 inhibition after 60 min (Table 2). Highly significant analgesic activity (63.33% inhibition) was observed after 120 min for the compound **3** as compared to reference compound, sulindac.

**Table 2.** Analgesic effect of drugs by Tail flick method in mice.

			Reaction time (s) post drug					
Treatment (*n* = 6)	Dose (mg/kg)	Reaction time (s) pre drug	30 m	% Inhibition	60 m	% Inhibition	120 m	% Inhibition
Compound **3**	150	5.00 ± 0.36	4.83 ± 0.30	3.33	7.33 ± 0.42**	46.66	7.16 ± 0.30***	63.33
Sulindac	100	4.83 ± 0.40	5.50 ± 0.34	13.79	8.83 ± 0.79***	82.75	11.00 ± 0.57***	127.58

All values represent mean ± SEM. ANOVA, followed by Dunnett’s multiple comparison test.

**p < 0.01. ***p < 0.001.

#### Hot plate method

Hot plate method was used for testing the analgesic activity. After 30 min, the % inhibition of test compound **3** was 15% as compared to reference drug sulindac with 41.30%.The testing compound **3** expressed highly significant activity of 67.50% inhibition compared to reference drug sulindac with 86.95 inhibition after 60 min (Table 3).

**Table 3.** Analgesic effect of drugs by Hot Plate method in mice.

			Reaction Time (seconds) post drug					
Treatment (*n* = 6)	Dose (mg/kg)	Reaction Time (seconds) pre drug	30 m	% Inhibition	60 m	% Inhibition	120 m	% Inhibition
Compound **3**	150	6.66 ± 0.33	7.66 ± 0.42	15	9.83 ± 0.30***	47.50	11.16 ± 0.30***	67.50
Sulindac	100	7.66 ± 0.33	10.83 ± 0.47***	41.30	11.83 ± 0.47***	54.34	14.33 ± 0.42***	86.95

All values represent mean ± SEM. ANOVA, followed by Dunnett’s multiple comparison test.

***p < 0.001.

#### Acetic acid –induced writhing

Acetic acid induced writhing was used for testing analgesic activity. The compound **3** expressed significant analgesic activity of 58.56% inhibition compared to reference drug sulindac with 74.1% inhibition (Table 4).

**Table 4.** Analgesic effect of drugs by acetic acid-induced writhing in mice.

Treatments (*n* = 6)	Dose (mg/kg)	Number of writhing in 20 min	% Inhibition
Compound **3**	150	14.66 ± 0.95***	56.86
Sulindac	100	8.83 ± 0.70***	74.01
Control (acetic acid)	0.1 ml of 20%	34.00 ± 1.23	—

All values represent mean ± SEM. ANOVA, followed by Dunnett’s multiple comparison test.

***p < 0.001.

#### Yeast-induced hyperthermia

Yeast-induced hyperthermia was used for testing analgesic activity in mice. The compound **3** expressed significant analgesic activity of after 120 min as compared to reference drug sulindac (Table 5).

**Table 5.** Effect of compound **3** and sulindac on yeast-induced hyperthermia in mice.

			Rectal temperature after yeast	Rectal temperature °C post drug administration		
Treatment (*n* = 6)	Dose (mg/kg)	Normal rectal temperature	administration 20 mL/kg of 20%	30 m	60 m	120 m
Compound **3**	150	35.16 ± 0.10	38.38 ± 0.19***	38.15 ± 0.10	37.83 ± 0.15*	37.33 ± 0.12***
Sulindac	100	35.21 ± 0.11	38.21 ± 0.31***	37.46 ± 0.14	37.16 ± 0.09**	36.23 ± 0.08***

All values represent mean ± SEM. ANOVA, followed by Dunnett’s multiple comparison test.

*p < 0.05, **p < 0.01, ***p < 0.001.

#### Anti-inflammatory activity

Based on the *in vitro* antioxidant activity, compound **3** was selected for *in vivo* anti-inflammatory activity by carrageenan induced paw edoema method. The anti-inflammatory activity of tested compound **3** after 3 and 5 h ranges from 50.52 to 50.54%, respectively compared to reference drug sulindac, which showed 65.18% after 3 h and 65.02% after 5 h (Table 6). Because of hydrazide substitution of *para* dimethylaminophenyl group, compound **3** presented significant anti-inflammatory activity.

**Table 6.** Anti-inflammatory activity of drugs by carrageenan-induced paw edoema method in albino rats.

		Before Carrageenan	Increase paw volume after 3 h			Increase paw volume after 5 h		
Group (*n* = 6)	Dose (mg/kg)	Mean ± SE	Mean ± SE	Net	% Inhibition	Mean ± SE	Net	% Inhibition
Only carrageenan		1.04 ± 0.02	1.68 ± 0.01***	0.63 ± 0.01		1.65 ± 0.01***	0.61 ± 0.02	Compound **3**
Compound **3**	150	1.02 ± 0.02	1.34 ± 0.02***	0.31 ± 0.03***	50.52	1.33 ± 0.01***	0.30 ± 0.02***	50.54
Sulindac	100	1.03 ± 0.03	1.26 ± 0.03**	0.22 ± 0.01***	65.18	1.25 ± 0.03***	1.25 ± 0.03***	65.02

All values represent mean ± SEM. ANOVA, followed by Dunnett’s multiple comparison test.

**p < 0.01, ***p < 0.001.

### The significant anti-inflammatory activity of compound 3 was observed

#### Ulcerogenic activity

The compound **3** was further evaluated for ulcerogenic and lipid peroxidation activity. Equimolar concentration of compound **3** and reference drug sulindac was administered as oral doses to the examined animals. Compound **3** demonstrated highly significant ulcerogenic reduction activity 4.33 ± 0.40 (40.90% inhibition) as compared to reference drug sulindac with 6.83 ± 0.40 (6.81% inhibition) (Table 7).

**Table 7.** Ulcer study of drugs using 80% Ethanol.

Treatments	Dose mg/kg	Ulcer Index	% Inhibition
80% Ethanol only	1 mL/200 g Rat	7.33 ± 0.33	
80% Ethanol only + Sulindac	100	6.83 ± 0.40	6.81
80% Ethanol only + Compound **3**	150	4.33 ± 0.4***	40.90
Sulindac only	100	1.33 ± 0.49	
Compound **3** only	150	—	

All values represent mean ± SEM. ANOVA, followed by Dunnett’s multiple comparison test.

***p < 0.001.

#### Ulcer study with indomethacin

Compound **3** was further evaluated for its ulcerogenicity as compared to indomethacin. Maximum ulcerogenic activity was observed in indomethacin (37.00 ± 1.59) as ulcer index. Sulindac (100 mg/kg) also produced the ulcer with ulcer index (32.00 ± 3.86) whereas compound **3** demonstrated a significant ulcerogenic reduction activity (22.16 ± 1.10) with 40.09% inhibition (Table 8).

**Table 8.** Ulcer study of drugs compared with Indomethacin

Treatments	Dose mg/kg	Ulcer Index	% Inhibition
Indomethacin	30	37.00 ± 1.59	
Sulindac only	100	32.00 ± 3.86	
Compound **3** only	150	22.16 ± 1.10***	40.09

All values represent mean ± SEM. ANOVA, followed by Dunnett’s multiple comparison test.

***p < 0.001.

Compound **3** with *para* dimethylaminophenyl substitution was found to be the most potent anti-inflammatory and analgesic derivative as well as a significant gastric sparing activity.

#### MDA, NP-SH, total protein content in gastric tissue

It has been known that the reduction of malondialdehyde (MDA) content in gastric tissue is consistent with the reduction of ulcerogenic activity. Compound **3** has shown a maximum reduction in the lipid peroxidation and gastric ulceration. The MDA content in compound **3** group was found to be (1.06 ± 0.02 nmol/g) as compared to the 80% ethanol group (6.53 ± 0.56 nmol/g) (Table 9).

**Table 9.** Anti-oxidant activity, MDA, NP-SH and total protein of drugs in stomach tissue of rat.

Treatments	Dose mg/dL	MDA (nmol/g)	NP-SH (nmol/g)	Total protein (g/L)
Normal saline	1 mL	1.03 ± 0.02	8.29 ± 0.53	113.37 ± 2.94
80% Ethanol only	1 mL	6.53 ± 0.56***	3.04 ± 0.39***	47.90 ± 2.89***
80% Ethanol only + Sulindac	1 mL + 100	2.28 ± 0.11***	5.08 ± 0.28**	93.81 ± 3.05***
80% Ethanol only + Compd. 3	1 mL + 150	1.59 ± 0.06***	6.62 ± 0.26***	100.99 ± 2.42***
Sulindac only	100	1.31 ± 0.04***	7.00 ± 0.032***	105.78 ± 1.43***
Compound **3** only	150	1.06 ± 0.02***	7.52 ± 0.31***	114.57 ± 1.89***

All values represent mean ± SEM. ANOVA, followed by Dunnett’s multiple comparison test.

***p < 0.001.

#### Toxicity of compound 3

Karber method was used to determine the LD_50_ of compound **3**. The LD_50_ of compound **3** was found to be 131 mg/kg (Table 10).

**Table 10.** LD_50_ determination of compound **3**.

Compound **3**	Group	Dose mg/kg	D. F (a)	Dead	M.M (b)	Pro. (a*b)
	1	100		0		
	2	200	100	0		
	3	400	200	1	0.5	100
	4	800	400	3	2	800
	5	1600	800	5	4	3200
	6	2000	400	9	7	2800
						6900
						1310
		Exp. Dose	131 mg/kg			

#### Effect of compound 3 on COX-2 mRNA expression in cisplatin induced hepatotoxicity in rats

**Figure 2.**
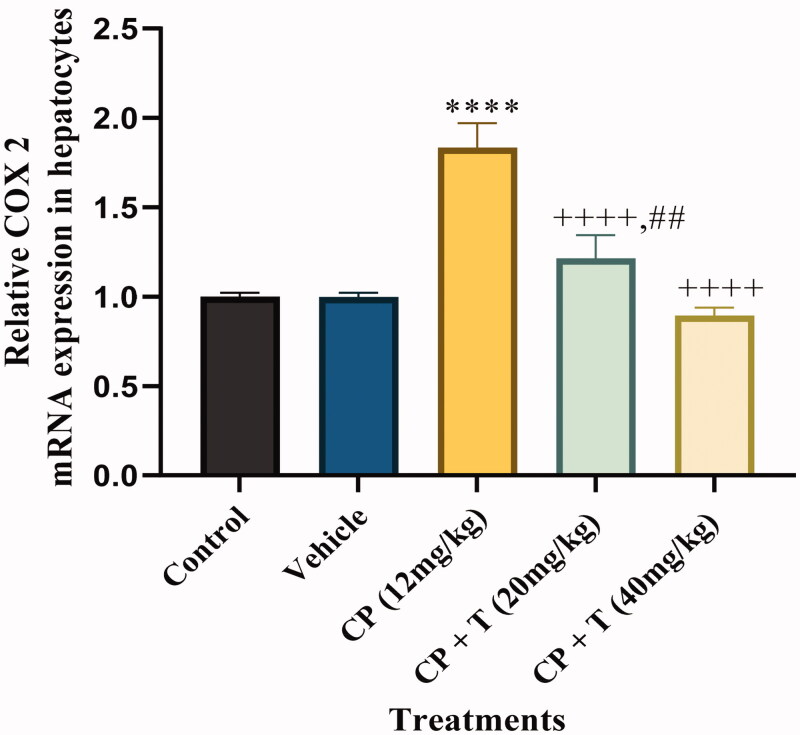
Quantitative real-time PCR analysis for COX-2 mRNA expression in hepatocytes of treated and untreated rats with actin as housekeeping gene. Data analysed by one-way analysis of variance using graph pad prism 8. Asterisks **** indicate *p* < 0.0001 significance from control and vehicle group, ^++++^ indicates *p* < 0.0001 significance from cisplatin (CP 12 mg/kg) treated group and ^##^ indicate *p* < 0.01 significance difference between CP + T (20 mg/kg) and CP + T (40 mg/kg) treatment groups. T stands for compound **3** treatment.

The expression of COX-2 is increased by pro-inflammatory meditaors^28^. Furthermore, the previous studies have revealed the increased COX-2 mRNA expression in cisplatin-induced hepatotocity^29^. Hence we characterised the effect of compound **3** administration on cisplatin-induced hepatotoxicity by measuring COX-2 mRNA levels in cisplatin-treated rats, untreated control rats, DMSO-treated rats, cisplatin–compound **3** treated (20 and 40 mg/kg) rats. Cisplatin‐induced hepatotoxicity was categorised by a significant increase in liver tissue gene expression of COX‐2 (*P* < 0.001), when compared to normal values. Co-treatment of cisplatin‐treated rats with compound **3** significantly downregulated liver tissue gene expression of COX‐2 (*P* < 0.001), as compared to the cisplatin values. Furthermore, co-treatment of cisplatin‐treated rats with 40 mg/kg dose of compound **3** significantly normalised liver COX‐2 gene expression (Figure 2).

#### Compound 3 administration induced reticence of COX-2 protein expression in liver tissue

**Figure 3.**
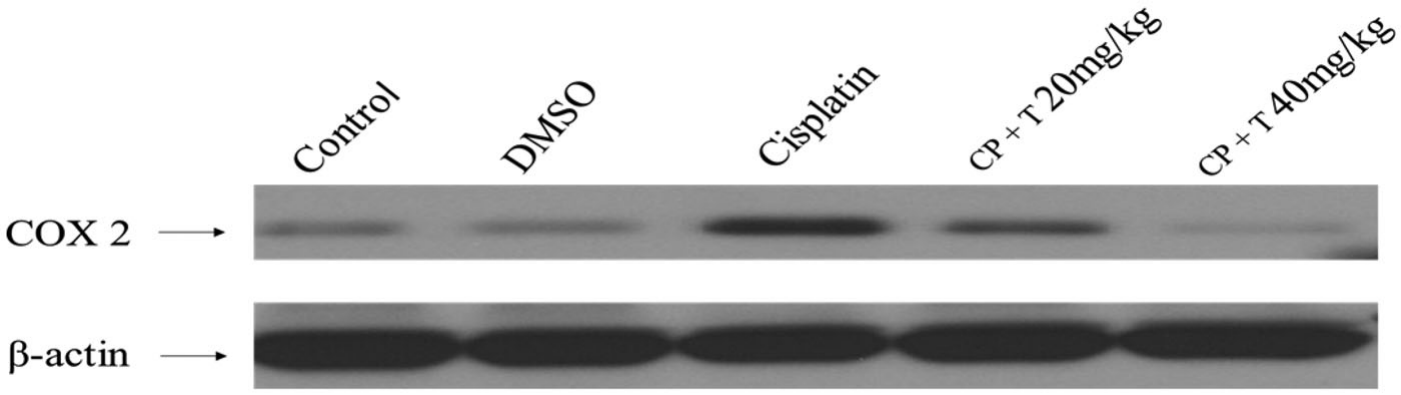
Effect of compound **3** treatment in regulating COX-2 protein expression in liver tissues of rats inoculated with cisplatin (12 mg/kg dose). Immunoblot analysis of COX-2 and β-actin in rat hepatocytes (*n* = 7). CP: cisplatin, T: compound **3** treatments.

We further examined the anti-inflammatory effect of compound **3** in liver tissues in cisplatin administrated rats by measuring protein expression of inflammatory mediators. In this regards, a substantial increase in COX-2 protein level was observed in the liver tissues isolated from cisplatin-administrated rats against untreated, control rats. The animals treated with compound **3** prior to cisplatin treatment resulted in a significant reduction in COX-2 protein expression when compared to cisplatin-treated group (Figure 3).

**Figure 4.**
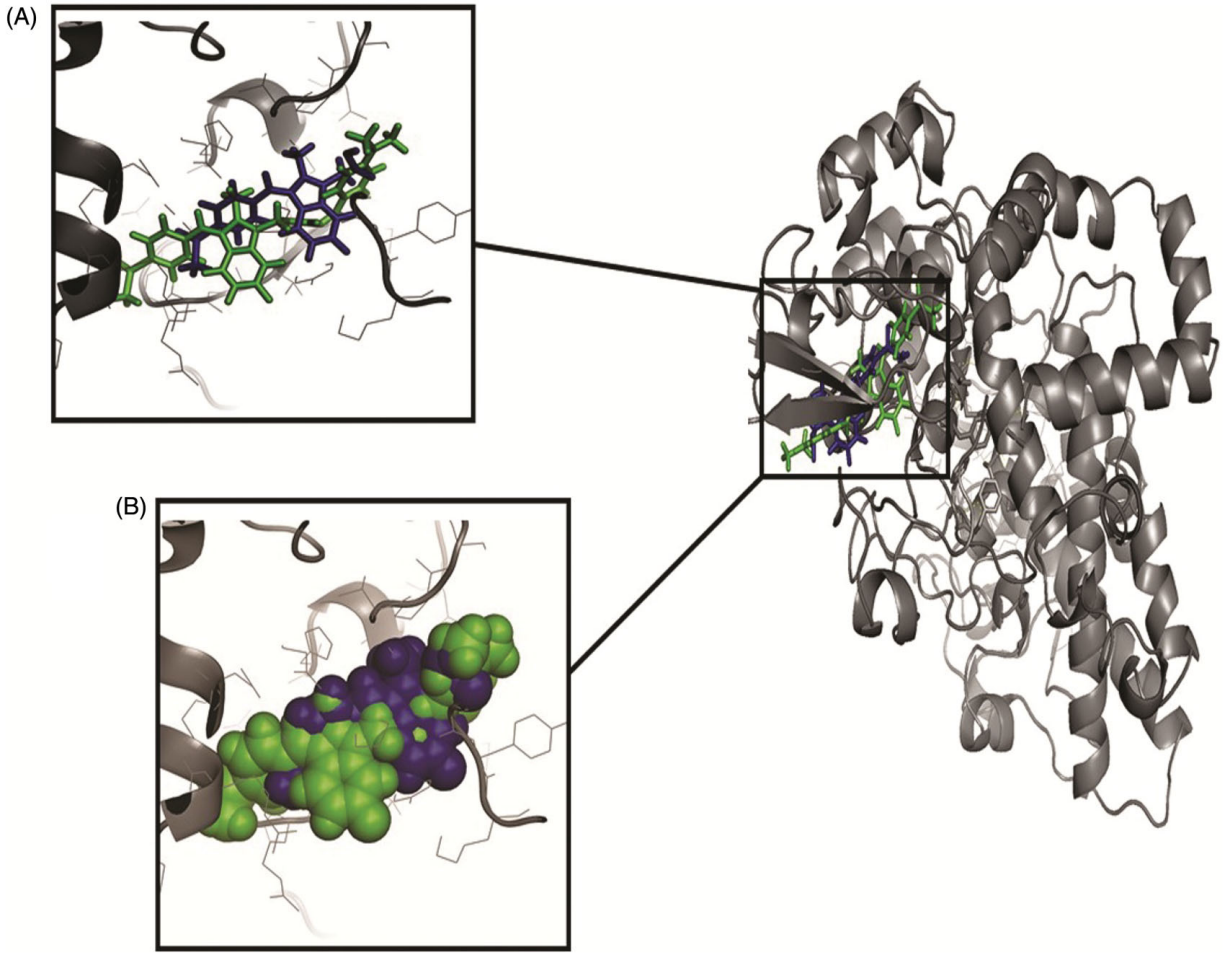
The orientation of docked compounds with Cox-2. The Sulindac is shown in blue colour while the compound **3** is shown in green colour. (A) The orientation of docked molecules is shown in sticks format. (B) The orientation of docked molecules is shown in spheres format.

**Figure 5.**
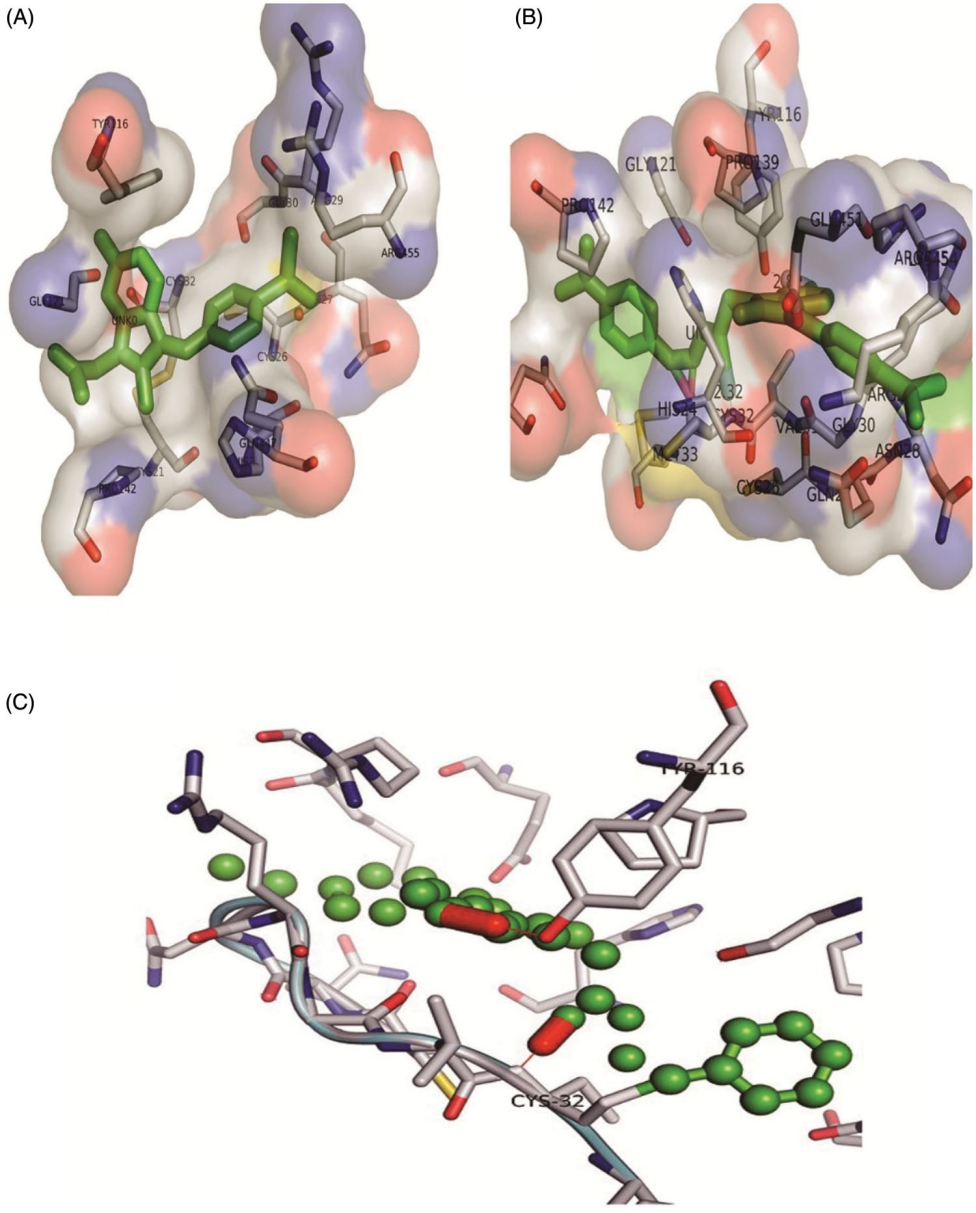
The orientation of Cox-2 residues making interactions with ligands (green) in 3 D confirmation. (A) The binding of drug Sulindac with Cox-2 protein. The amino acids residues are making hydrophobic interaction with drug. (B) The binding of compound **3** with Cox-2 protein. The amino acids residues are making two hydrogen bonds and hydrophobic interaction with the compound. (C) The Cys-32 and Tyr-116 making hydrogen bonds with compound **3**.

**Figure 6.**
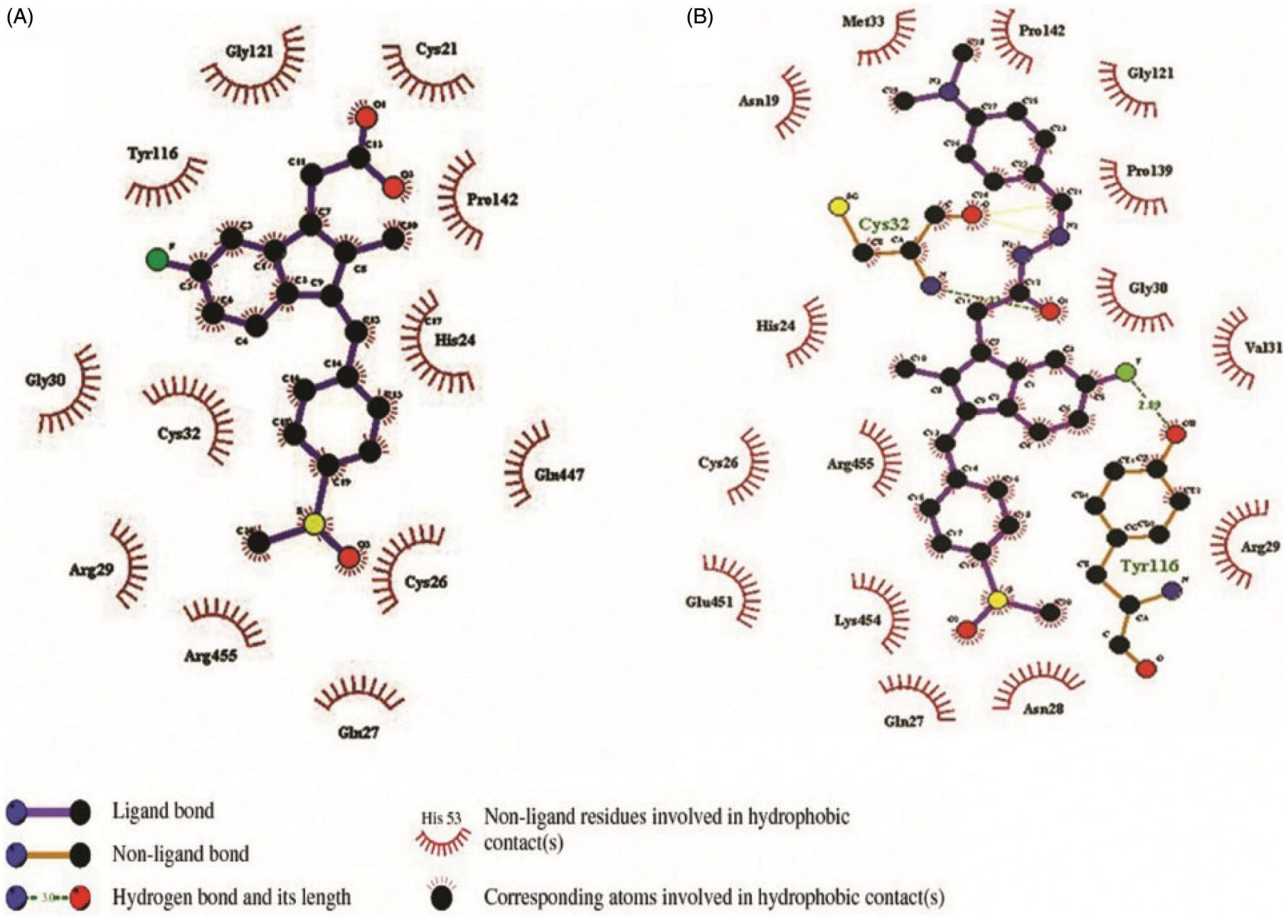
2D map of interactions of Cox-2 protein with Sulindac (A) and compound **3** (B).

The binding affinities of all the synthesised compound **(1 – 25)** and Sulindac with Cox-2 were predicted using geometry-based molecular docking. The binding affinities of the compounds were demonstrated by PatchDock server with ACE values (Table 11). The lower ACE value is considered to be associated with better ligand affinity with the enzyme^30^. Compound 3 showed lower ACE values (−440.29 kJ/mol) as compared to the standard drug Sulindac (−325.99 kJ/mol). This data suggests a better binding affinity of the compound 3 with Cox-2 protein as compared to the Sulindac (Figure 4). The visualisation of the docked complex revealed two hydrogen bonds by compound 3 with Cys-32 and Tyr-116, along with many hydrophobic interactions (Figure 5). While Sulindac showed only the hydrophobic interaction with the protein in the docked complex (Figure 6). It can be speculated that the formation of hydrogen bonds by compound 3 is responsible for the better affinity of the compound as compared to Sulindac.

**Table 11.** Docking energies of compounds **(1–25)** and sulindac.

Compound	Docking energies	Compound	Docking energies
**1**	−81.19	**14**	−247.54
**2**	−257.54	**15**	−298.94
**3**	−440.29	**16**	−252.92
**4**	−417.10	**17**	−266.51
**5**	−413.27	**18**	−227.18
**6**	−96.75	**19**	−220.75
**7**	−392.71	**20**	−150.57
**8**	−317.14	**21**	−220.38
**9**	−388.61	**22**	−457.75
**10**	−345.55	**23**	−421.83
**11**	−242.36	**24**	−466.48
**12**	−428.94	**25**	−77.10
**13**	−275.16	Sulindac	−325.99

## Conclusion

In conclusion, novel sulindac hydrazide derivatives **(1–25)** were synthesised in good yields and characterised by spectral data and elemental analysis. Chemical modification to the sulindac acetohydrazide scaffold resulted in twenty-five derivatives with significant antioxidant activity. Compound **3** containing *para* dimethylaminophenyl group was found to be having highly potent antioxidant activity. It was evaluated for *in vivo* anti-inflammatory and various analgesic and ulcerogenic activity different animal models and was found to be significant anti-inflammatory and analgesic agent. It demonstrated significant ulcerogenic reduction activity in ethanol and indomethacin model. The LD_50_ of compound **3** was found to be 131 mg/kg. Compound **3** with *para* dimethylaminophenyl substitution was found to be the most potent anti-inflammatory and analgesic derivative as well as a significant gastric sparing agent. Compound **3** significantly downregulated liver tissue gene expression of COX‐2. The animals treated with compound **3** prior to cisplatin treatment resulted in a significant reduction in COX-2 protein expression when compared to cisplatin-treated group.

## Data Availability

Samples of the compounds **(1‒25)** in pure form are available from authors.
